# Global Autozygosity Is Associated with Cancer Risk, Mutational Signature and Prognosis

**DOI:** 10.3390/cancers12123646

**Published:** 2020-12-04

**Authors:** Limin Jiang, Fei Guo, Jijun Tang, Shuguan Leng, Scott Ness, Fei Ye, Huining Kang, David C. Samuels, Yan Guo

**Affiliations:** 1 Department of Internal Medicine, Comprehensive Cancer Center, University of New Mexico, Albuquerque, NM 87109, USA; lijiang@salud.unm.edu (L.J.); sleng@salud.unm.edu (S.L.); sness@salud.unm.edu (S.N.); HuKang@salud.unm.edu (H.K.); 2 School of Computer Science and Technology, College of Intelligence and Computing, Tianjin University, Tianjin 300350, China; fguo@tju.edu.cn; 3 Department of Computer Science, University of South Carolina, Columbia, SC 29208, USA; jtang@cse.sc.edu; 4 Department of Biostatistics, Vanderbilt University, Nashville, TN 37232, USA; fei.ye@vumc.org; 5 Department of Molecular Physiology and Biophysics, Vanderbilt Genetics Institute, Vanderbilt University, Nashville, TN 37232, USA; david.c.samuels@vanderbilt.edu

**Keywords:** global autozygosity, runs of homozygosity, heterozygosity ratio, mutational signature, prognosis

## Abstract

**Simple Summary:**

Global autozygosity in the form of runs of homozygosity is associated with various diseases. Heterozygosity ratio, an alternative measure of global autozygosity, is used to assess cancer risk in this study. Our analysis shows strong and consistent associations between heterozygosity ratios and various cancer types. Further analysis reveals the heterozygosity ratio’s potential connections to mutational signatures and cancer prognosis.

**Abstract:**

Global autozygosity quantifies the genome-wide levels of homozygous and heterozygous variants. It is the signature of non-random reproduction, though it can also be driven by other factors, and has been used to assess risk in various diseases. However, the association between global autozygosity and cancer risk has not been studied. From 4057 cancer subjects and 1668 healthy controls, we found strong associations between global autozygosity and risk in ten different cancer types. For example, the heterozygosity ratio was found to be significantly associated with breast invasive carcinoma in Blacks and with male skin cutaneous melanoma in Caucasians. We also discovered eleven associations between global autozygosity and mutational signatures which can explain a portion of the etiology. Furthermore, four significant associations for heterozygosity ratio were revealed in disease-specific survival analyses. This study demonstrates that global autozygosity is effective for cancer risk assessment.

## 1. Introduction

The human genome is comprised of approximately three billion base pairs. Single Nucleotide Polymorphisms (SNPs) can affect various disease risks as shown by numerous genome-wide association studies (GWAS). According to the GWAS catalog (May 2020), 4424 unique SNPs have been found to influence cancer risk with *p* < 10^−5^ significance. While an SNP describes the allelic information at a single genomic position, global heterozygosity and homozygosity describe the genome-wide zygosity level. Heterozygosity describes the possession of two different alleles of an SNP, and homozygosity describes the possession of the same allele at a genomic position. Global homozygosity is often measured in the form of Runs of Homozygosity (ROH) [1], which is a measure of the segments of the genome without heterozygous SNPs. The associations between ROH and many phenotypes have been thoroughly established, such as height [2], schizophrenia [3], Alzheimer’s Disease [4], along with many others. ROH can be calculated through common genomic toolboxes, such as PLINK [5] and BCFtools [6]. The units of ROH can vary. In some studies [2,3,4], the number of ROH segments detected (based on a minimum threshold) was used for the association study. In another [7], the median length of ROH was used. Regardless, the computation of ROH is dependent on many factors, such as the density and quality of SNP data, the number of tolerated heterozygous SNPs within an ROH, and the size of the sliding window. It has been demonstrated that ROH is highly sensitive to SNP density (the coverage of genome-wide SNPs by an array) and that genotyping arrays of different SNP densities may produce very different ROH results [7]. These inconsistencies in ROH computation can lead to contradictions in ROH association analyses [8,9,10]. 

An alternative measure of autozygosity is the Heterozygosity Ratio (HR), which is the ratio between the number of heterozygous SNPs and the number of non-reference homozygous SNPs. The HR was originally proposed as a quality control parameter for SNP data because it has a theoretically expected value of 2 [11]. A subsequent study showed that the observed average HR value is dependent on race, with only African ancestry individuals having a ratio close to 2, whereas the HRs for other races are substantially lower in empirical data [12]. Unlike ROH, the computation of HR does not require adjustable parameters. Thus, HR is more robust than ROH against variable SNP density [7]. ROHs have previously been tested for cancer risk; most of those studies found no association [13,14]. HR’s association with cancer risk has not been evaluated. In this study, we focused on global HR measures and their associations with cancer risk, cancer prognosis, and the mutagenesis process.

## 2. Results

### 2.1. HR_NonRef_ vs. HR_Minor_ vs. ROH

**Figure 1:**
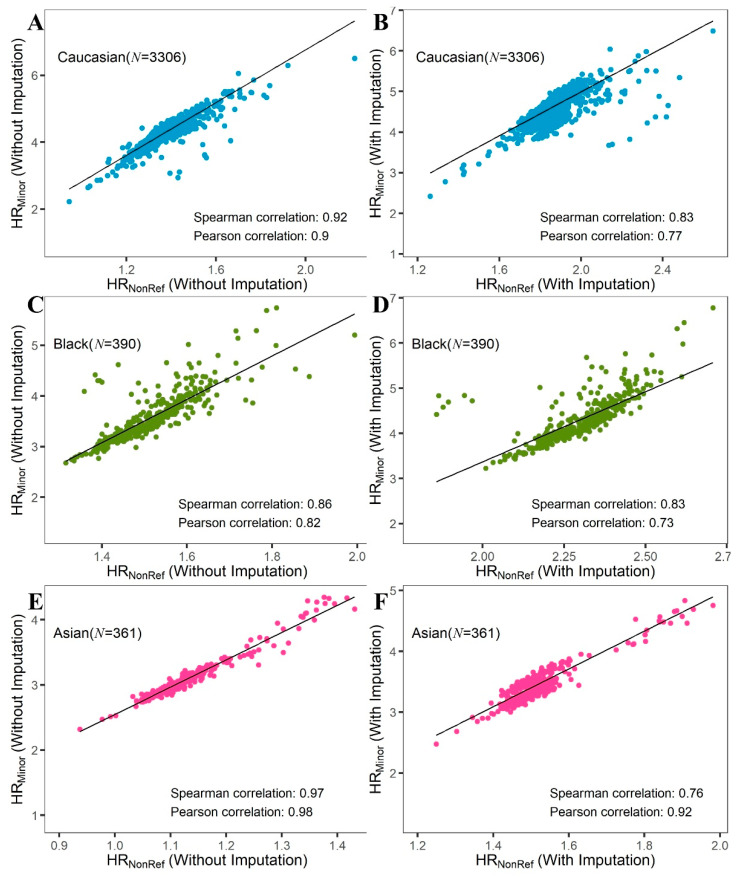
Scatter plots for HR_NonRef_ vs. HR_Minor_. (**A**,**B**): Caucasian; (**C**,**D**): Black; (**E**,**F**): Asian. (**A**,**C**,**E**): Heterozygosity Ratio (HR) computed from original Single Nucleotide Polymorphism (SNP) data without imputation. (**B**,**D**,**F**): HR computed from SNP data after imputation. Each data point is an individual in the cohort. All results show excellent correlations between HR_NonRef_ and HR_Minor_, with HR_Minor_ two to three times higher than HR_NonRef_.

The HR was traditionally computed as the ratio between the number of heterozygous SNPs divided by the number of non-reference homozygous SNPs, which we define as HR_NonRef_. Since the human reference genome was constructed from a small subset of individuals, it is limited by a small sample size and poor global race representation. These limitations result in many cases where the reference allele is not the major allele of the population. By comparing The Cancer Genome Atlas (TCGA) SNP data and reference allele in the GRCh38 genome, we estimated that around 8% of the variants in the human reference genome may not represent the major allele of the population. This inconsistency between reference allele and major allele has a potential effect on HR computation, which has not been previously considered. To investigate this potential difference, we define HR_NonRef_ and HR_Minor_, where HR_NonRef_ uses reference alleles based on the traditional HR definition and HR_Minor_ uses major alleles defined by the study cohort instead. HR_NonRef_ and HR_Minor_ were computed for all TCGA SNP and International Genome Sample Resources (IGSR) SNP datasets. High correlations were observed between HR_NonRef_ and HR_Minor_ for all Caucasians, Blacks, and Asians (Figure 1), although the correlation dropped slightly after imputation, most likely due to the additional noise introduced. The only difference between HR_NonRef_ and HR_Minor_ is the scaling, as HR_Minor_ appears to be 2 to 3 times the value of HR_NonRef_. Due to the similarity between HR_NonRef_ and HR_Minor_, we chose to use HR_NonRef_ for all subsequent analyses. Furthermore, the scatter plot (Figure S1) between HR_NonRef_ and HR_Minor_ resembles the scatter plot of principal component 1 vs. principal component 2 from a principal component analysis of ancestry informative markers. This result implies that the differences in HR measures between the two definitions is strongly associated with race.

**Figure 2:**
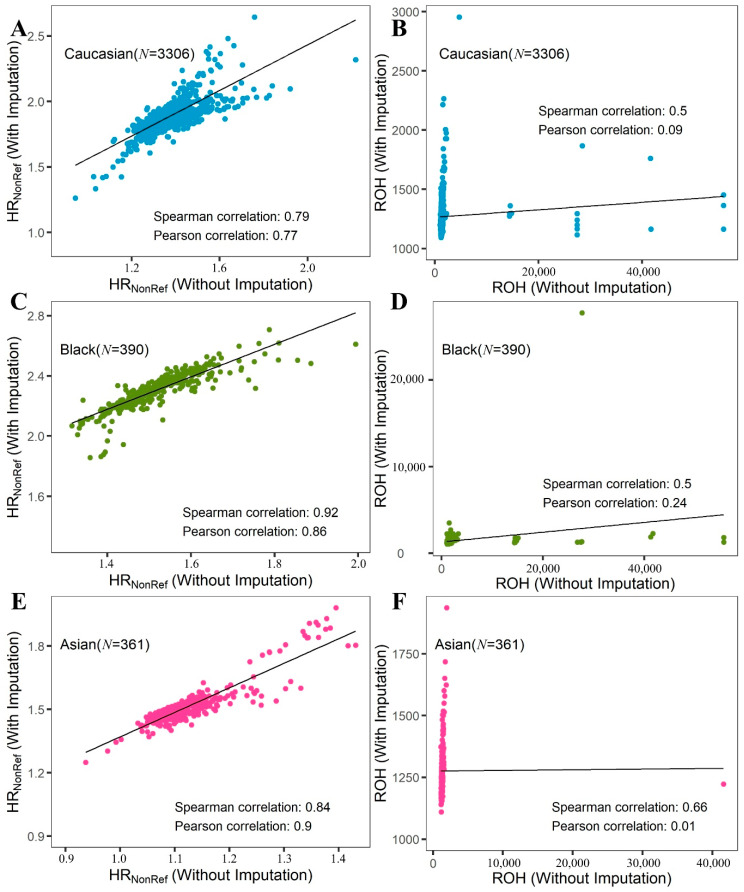
Scatter plot for HR_NonRef_, Runs of Homozygosity (ROH), before and after imputation. (**A**,**B**): Caucasian; (**C**,**D**): Black; (**E**,**F**): Asian. (**A**,**C**,**E**): HR_NonRef_; (**B**,**D**,**F**): ROH. The correlations for ROH are weaker than HR_NonRef_ before and after imputation due to the ROH outliers resulted from imputation. This shows that HR is less prone to the effect of SNP density than ROH.

**Figure 3:**
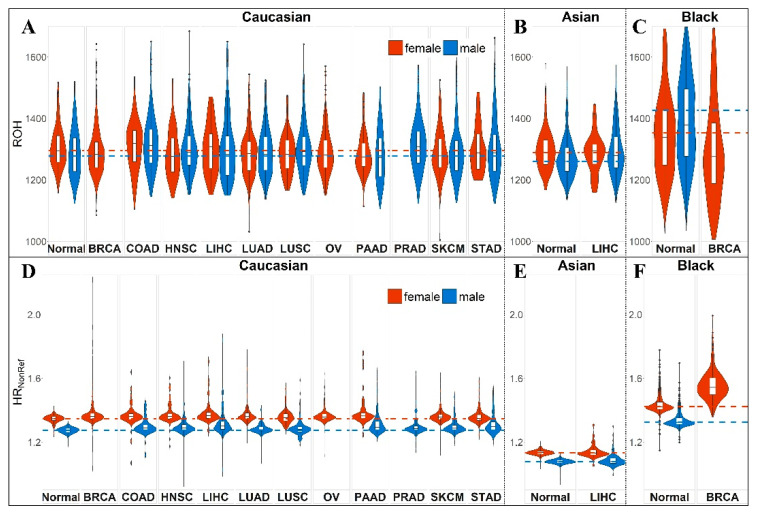
Comparison of HR_NonRef_ and ROH between sex across all three races tested. The computation of HR_NonRef_ and ROH contains sex chromosomes X and Y. For the equivalent of this figure without chromosomes X and Y, please see Figure S2. (**A**) Violin and boxplots of ROH separated by sex for Caucasians. (**B**) Violin and boxplots of ROH separated by sex for Asians. (**C**) Violin and boxplots of ROH separated by sex for Blacks. (**D**) Violin and boxplots of HR_NonRef_ separated by sex for Caucasians. (**E**) Violin and boxplots of HR_NonRef_ separated by sex for Asians. (**F**) Violin and boxplots of HR_NonRef_ separated by sex for Blacks. Females, in general, have higher HR_NonRef_ than males and the difference is substantially more visible than ROH.

A previous study [7] has shown that HR is more robust than ROH due to immunity to SNP density. We performed verification of this result by comparing the results before and after imputation. Figure 2 shows that imputation produced ROH outliers which severely hampered the overall correlation of ROH. We further evaluated the race and sex differences between HR and ROH. Differences by race for HR_NonRef_ and ROH are shown in Figure 3A, with the highest average HR for Blacks, followed by Caucasians and Asians. These results are consistent with previous publications [7,12]. Sex differences for HR were not previously studied. For HR_NonRef_, females have a substantially higher HR than males for all races (Figure 3A). For ROH, females have higher ROH than males in Caucasians (Figure 3A) and Asians (Figure 3B), males had higher ROH than females in Blacks (Figure 3C). However, the sex difference of HR and ROH is primarily contributed by the sex chromosomes, and after removing Chromosomes X and Y from the computation of HR and ROH, the difference is substantially reduced (Figure S2). The violin plots also demonstrate that HR_NonRef_ has less variation than ROH. The cancer subjects’ median HR_NonRef_ is visibly higher than normal subjects’ median HR_NonRef_, which results in significant associations with cancer risk. Furthermore, we found no significant association between HR_NonRef_/ROH and age, which is in concordance with the conventional genetic concept that that germline variants should not be affected by age. Because of the strong differences of HR_NonRef_ and ROH based on sex and race, and varying prevalence of cancer, all subsequent analyses were stratified by sex and race. 

### 2.2. Global Autozygosity and Cancer Risk

We evaluated the association of HR_NonRef_ and ROH with cancer risk using logistic regression with cancer cases from TCGA and healthy controls from IGSR. To avoid any selection bias, HR_NonRef_ and ROH were computed from SNPs present in both TCGA and IGSR. Races (Caucasian, Black and Asian) and sex were tested separately. Tests were limited to case sample size greater than 100. Ten cancer types met the criteria and were tested. For Caucasians, logistic regression analyses showed that HR_NonRef_ is significantly positively associated with cancer risk in all ten cancer types (Table 1). The strongest cancer association was for male skin cutaneous melanoma ($p=5.28\times 10^{-12})$, followed by female ovarian cancer $\left(p=3.34\times 10^{-11}\right)$. For Blacks, only breast invasive carcinoma met the case sample size greater than 100 criteria. HR_NonRef_ was found to be positively significantly associated with breast invasive carcinoma ($p=4.89\times 10^{-28})$, a result more extreme than the Caucasian’s counterpart. For Asians, only liver hepatocellular carcinoma met the sample size requirement, and HR_NonRef_ was positively significantly associated (p=0.001). The global positive associations between HR_NonRef_ and cancer risk suggest that individuals with a more heterozygous genome are at higher risk for multiple cancer types. Receiver operating characteristic (ROC) curves show that statistically significant cancer, race, sex groups had area under curve (AUC) between 0.54 and 0.88, with Black females in breast cancer being the most predictive group (Figure S3).

For Caucasians, ROH was found to be significantly associated with nine out of ten cancer types (Table 2). However, the directions of association were mixed, with four negative and five positive associations. Furthermore, there were several differences based on sex. In head and neck squamous cell carcinoma, ROH was not significantly associated with cancer risk for females but was nominally significant for males (p=0.02). In lung adenocarcinoma, ROH was significantly associated with cancer risk for females (p=0.007), but was not significant for males. Similarly, in skin cutaneous melanoma, ROH was nominally significantly associated with cancer risk for females (p=0.01), but not for males. For Blacks, in breast invasive carcinoma, ROH was borderline associated with breast invasive carcinoma (p=0.07). For Asians, in liver hepatocellular carcinoma, ROH was significantly associated with cancer risk for males (p=0.03). The associations between HR_NonRef_ and cancer risk are stronger and more consistent than those of ROH. The inconsistency of association direction and the inconsistency between sexes observed in ROH results may also represent the instability of ROH measurement from incomplete genotyping data. 

**Figure 4:**
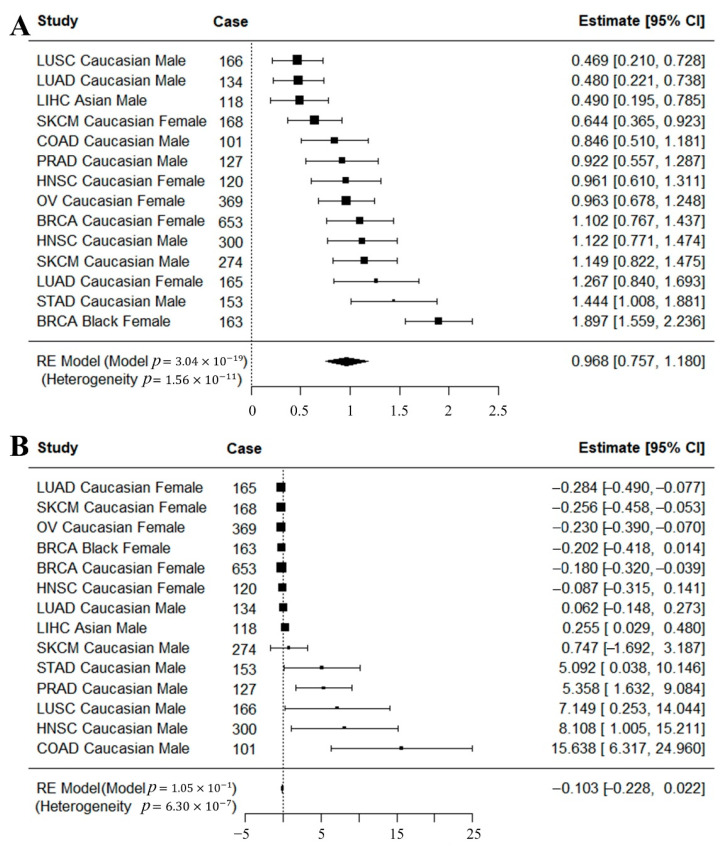
Meta-analysis for cancer risk. (**A**) Meta-analysis of cancer risk results of HR_NonRef_ (Table 1). (**B**) Meta-analysis of cancer risk results of ROH (Table 2). Heterogeneity *p* < 0.05 indicates significant heterogeneity across cancer datasets, thus a random effect model was used for the meta-analysis. HR_NonRef_ behaved consistently across multiple cancer types, which resulted in a significant meta-analysis *p*-value. ROH, on the other hand, was not significant.

In addition to the cancer risk analysis by race, sex and cancer type, we also conducted meta analysis across all possible cancer types to study the overall effect. The meta-analyses were conducted based on the results from cancer risk analysis with the same inclusion criteria. A random effect model was used because the heterogeneity test was significant which indicated heterogeneity across the cancer types. A meta-analysis on HR_NonRef_ showed significant association of HR_NonRef_ with cancers ($p=3.04\times 10^{-19}$) (Figure 4A). For ROH, the meta random effect model produced was not significant (p=0.1) (Figure 4B). The meta analysis further demonstrated the robustness of HR_NonRef_ over ROH.

### 2.3. Mutational Signatures and Survival Analysis

Somatic mutation is one of the most important aspects of cancer. Somatic mutations occur as the consequence of a mutational process that is triggered by either endogenous errors in DNA replication and repair or exogenous mutagens. Taking into consideration DNA’s complementarity, six distinct substitutions can be formed between the four nucleotides. When adding up the 5’-neighbor and the 3’-neighbor, we can derive a three-nucleotide motif from the focal substitution, and thus expand the six-substitution inventory to a 96-motif catalog, known as mutational signatures [15]. The profile of various mutational motifs in a cancer patient can be modeled as a combination of distinct mutational signatures. A mutational signature is conceived as the footprint of a mutational process in the nuclear genome, represented in the form of relative frequencies of the motifs of a mutational catalog [16,17,18,19,20]. 

Both mutational signature and global autozygosity represent genome-wide patterns, with mutational pattern at the somatic level and HR and ROH at the underlying germline level. As we have shown that HR_NonRef_ is highly associated with cancer risk, we hypothesize that HR_NonRef_ may be related to mutational signatures. Using TCGA somatic mutation data, we fit each patient into the established COSMIC reference mutational signatures, as described in the Methods section. Linear regression models were used to describe the association between mutational signatures and HR_NonRef_ and ROH. False discovery Rate (FDR) < 0.05 was used as the significant threshold. Datasets with a sample size greater than 100 were included in the analyses. Eleven significant associations were identified, seven for HR_NonRef_ and four for ROH (Table 3). All 11 significant results were from those subjects of Caucasian descent. Five of the seven significant HR_NonRef_ associations were from the ovarian cancer dataset, and consisted of SBS9 (FDR=0.001), SBS18 (FDR=0.001), SBS5 (FDR=0.007), SBS7c (FDR=0.007), and SBS22 (FDR=0.03). SBS9 is a mutational signature resulting from mutations during replication by polymerase eta. SBS18’s etiology is proposed to be damaged by reactive oxygen species; SBS5’s etiology is currently unknown; SBS7c is related to ultraviolet light damage and is possibly the consequence of translesion DNA synthesis by enzymes with a propensity to insert T, and SBS22 is related to aristolochic acid exposure. The other two significant associations with HR_NonRef_ were SBS44 (related to DNA mismatch repair, FDR=0.02) in female skin cutaneous melanoma and SBS36 (related to defective base excision repair, FDR=0.045) in prostate adenocarcinoma. The most significant association was found between ROH and SBS44 $\left(FDR=4.62\times 10^{-8}\right)$ in females with head and neck squamous cell carcinoma. The other three significant associations with ROH were SBS36 ($FDR=3.02\times 10^{-5})$ in prostate adenocarcinoma, SBS42 (related to haloalkanes exposure, FDR=0.0002) in male lung squamous cell carcinoma, and SBS7b (related to ultraviolet light exposure, FDR=0.02) in males in male lung squamous cell carcinoma. 

**Figure 5:**
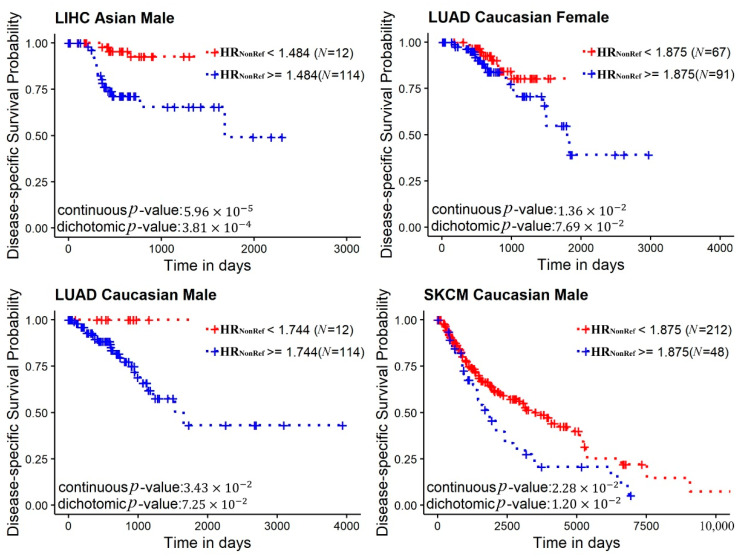
Disease-specific survival analysis results presented in Kaplan–Meier plots. R’ maxstat package was used to identify the optimal point for dichotomization. Two *p*-values are presented in the figure, one from Cox proportional hazards regression models without dichotomizing the data, and one from Cox proportional hazards regression models with dichotomized data.

Next, we performed survival analyses to examine whether HR_NonRef_ and ROH have prognostic value. Disease-specific survival analyses using Cox proportional hazard models identified no significant results for ROH under any scenarios. For HR_NonRef_, four race and gender-specific significant results were found (Figure 5): Asian males and liver hepatocellular carcinoma ($p=5.96\times 10^{-5})$, Caucasian males and lung adenocarcinoma (p=0.03), Caucasian females and lung adenocarcinoma (p=0.01), and Caucasian males and skin cutaneous melanoma (p=0.02). All survival results show that lower HR_NonRef_ is associated with better prognosis.

## 3. Discussion

A single SNP can have a severe impact, as shown by Mendelian diseases. Multiple SNPs together can help explain a portion of a disease’s variation in the population but never fully account for the heritability. This is the famous missing heritability problem [21]. One proposed solution for this problem is that a person’s susceptibility to disease may be polygenic and dependent on many low effect variants [22]. Global autozygosity measurement expands on the polygenic idea, by measuring the genome globally. Global autozygosity as a risk factor for diseases such as schizophrenia and Alzheimer’s have been established. However, its connection to cancers has not been examined previously. 

Cancer risk analysis showed that both HR_NonRef_ and ROH are closely associated with cancer risk. However, given the same sample size, HR_NonRef_ demonstrated stronger associations than ROH. Eight of the 14 significant HR_NonRef_ associations were at ≤10^−8^, the GWAS significance level. However, we stress that since we did not carry out 1 million independent tests, GWAS level significance is not required for the multiple testing correction. For ROH, the most significant association is at *p* = 0.0009. Furthermore, the associations between HR_NonRef_ and cancer risk are more consistent than ROH. While all 14 significant HR_NonRef_ and cancer risk associations are positive, eight of the 14 significant associations for ROH are positive, and six are negative. These results further illustrate the robustness of HR as a characterization of the genome variability.

The literature [23,24] has shown that a single SNP can increase cancer risk. Our analysis results for global autozygosity also suggest that genome-wide characteristics can also affect cancer risk. However, the etiology behind global autozygosity and cancer risk is not well understood, and it would be even harder to study the etiology for global autozygosity compared to single SNPs due to the lack of precise targets. Nonetheless, we performed additional analyses to assess the associations between global autozygosity and mutational signatures. Mutational signatures are constructed from somatic mutations, which can represent the mutagenesis history. A previous study has shown the link between germline variants and somatic mutation [25]. For example, germline variants in *RBFOX1* increased the incidence of *SF3B1* somatic mutation eight-fold via functional alterations in RNA splicing, and 19p13.3 variants were associated with a four-fold increased likelihood of somatic mutations in *PTEN*. Thus, it is not unreasonable to hypothesize that there are connections between global autozygosity and mutational signatures. Our analyses found eleven significant associations between global autozygosity and various mutational signatures after correcting for false discovery. These results suggest that global autozygosity is related to some mutational processes. It might affect the risk of DNA mismatch in the repair process after exposure to carcinogens such as ultraviolet light and haloalkanes. Disease-specific survival analysis also identified four significant associations for HR_NonRef_, which also suggest potential prognosis associations of global autozygosity. 

One of the limitations of HR_NonRef_ is that it requires the measurement of the entire genome. Compared to biomarkers of a few SNPs and genes, HR_NonRef_ computation is more expensive and time consuming. However, in previous work [7], we showed that HR_NonRef_ computed from a random subset of SNPs can be a robust representation of the true HR_NonRef_. Furthermore, the price of whole genome genotyping has dropped below USD 100 per subject, well within the range of acceptable cost. Further cost reduction can be achieved by estimating HR_NonRef_ from the subset of SNPs. Although, the criteria of the subset of SNPs to best estimate HR_NonRef_ requires additional study. 

## 4. Materials and Methods 

### 4.1. Genotyping Data Acquisition and Imputation

Germline SNP data were obtained from 4833 subjects with 12 cancer types from the Affymetrix Genome-Wide Human SNP Array 6.0 in The Cancer Genome Atlas (TCGA), which contains 934,968 SNPs. All SNP data used in this analysis were derived from blood samples. Additional SNP imputation was performed using a Hapmap Phase 3.0 reference through the Michigan Imputation Server [26]. Imputed SNPs with R^2^ > 0.8 were retained for further analysis. After imputation, each cancer type contained 10–16 million SNPs. The total SNP number was 164,497,868. SNP data with imputation of 1668 subjects from The International Genome Sample Resources (IGSR), formally known as the 1000 Genome Project, were also downloaded.

### 4.2. Somatic Mutation Data Acquisition and Mutational Signature Computation

Somatic mutation data of 10,179 patients with 33 cancer types were downloaded from the Genomic Data Commons, the gateway of TCGA. The cancer type abbreviations, full name, and detailed sample size are available in Table S1. The probability matrix for 49 established COSMIC reference mutational signatures (v3) was downloaded from Synapse Documentation (https://www.synapse.org/#!Synapse:syn11738319) (Table S2). We formalized a catalog of 96 three-nucleotide motifs that surround the mutational focus (one upstream nucleotide, one mutation site, and one downstream nucleotide site), and derived frequency tables of this motif catalog for each patient. We leveraged a computational function from the R package MutationalPatterns [27] to fit the patient mutational motif frequency tables to the reference mutational signatures while requiring the coefficients, i.e., signature-to-patient contribution strengths, to be non-negative values. The estimated coefficients formed a 96-by-10,179 matrix of non-negative values, representing the distribution of 96 mutational motifs across the 10,179 patients.

### 4.3. HR and ROH Computation

Two types of HR were computed, which we denote as HR_NonRef_ and HR_Minor_. HR_NonRef_ is computed as N_het_/N_HomNonRef_, where N_het_ is the number of heterozygous SNPs, and N_HomNonRef_ is the number of homozygous non-reference SNPs. These definitions are consistent with previous studies [7,11,12] of HR. To study the potential effect when the reference allele does not equal the major allele in a cohort, we also defined HR_Minor_ as N_het_/N_HomMinor_, where N_HomMinor_ is the number of homozygous minor alleles based on the patient cohort. ROHs were computed using PLINK [5]. The median ROH length was used for subsequent analyses. 

### 4.4. Statistical Analyses

All statistical analyses were conducted using 64 bit R 4.0.2. Both Spearman’s and Pearson’s correlation coefficients were used to compare HR_NonRef_ and HR_Minor_. Linear regression (R glm function with family = Gaussian parameter) was used to evaluate the association between age and HR_NonRef_. Logistic regression (R glm function with family = binomial parameter) was used to evaluate the association between HR/ROH and cancer risk. The cancer cases from TCGA were matched with non-cancer controls from IGSR by race and sex. Moreover, to avoid any potential bias, the common set of SNPs between TCGA and IGSR was used to compute HR and ROH. The unit for HR_NonRef_ and ROH was per standard deviation. The R function coxph was used to evaluate the survival predictability of HR_NonRef_ and ROH. Both HR_NonRef_ continuous and dichotomized models were conducted. For the dichotomized model, the R package maxstat was used to find the optimal dichotomization threshold. This threshold was used for dichotomizing HR_NonRef_ into high and low groups for the Kaplan–Meier curve presentation. The associations between mutational signatures and HR_NonRef_/ROH were found by linear regression (R glm function with family = Gaussian parameter). The R function p.adjust with FDR parameter was used for adjusting for multiple test correction. ROC curves were drawn with the ggplot2 package. The auc_roc function from mltools R package was used to compute AUC.

Meta-analyses across all possible cancer types and groups were conducted for HR_NonRef_ and ROH. The meta-analyses were conducted based on the results from cancer risk analysis (Table 2 and Table 3). The R function rma from the metafor package was used for the meta analysis. The random effect model was used during the meta analysis because the heterogeneity test across cancer types was significant (*p* < 0.05). 

## 5. Conclusions

Our analyses of global autozygosity show that HR_NonRef_ is a more robust measurement than ROH. More importantly, our study demonstrates the connections between global autozygosity and cancer risk. We identified strong associations between HR_NonRef_ and cancer risk. Even though the majority of the subjects were Cacuasian, strong associations for minority groups, such as breast invasive carcinoma risk in Black women and liver hepatocellular carcinoma in Asian men, were identified. Further evidence was identified by exploring the associations between global autozygosity, mutational signatures, and cancer prognosis. These results show that global autozygosity can be used for reliable cancer risk assessment. 

## Figures and Tables

**Table 1:** Results from logistic regression assessing HRNonRef’s cancer risk.

LogOR ^1^ (95% CI)	*p*	Cancer ^2^	Race	Gender	Cases	Controls ^3^
1.8973 (1.6228–2.1920)	4.89 × 10^−28^	BRCA	Black	female	163	342
1.1487 (0.8858–1.4336)	5.28 × 10^−12^	SKCM	Caucasian	male	274	240
0.9630 (0.7327–1.2104)	3.34 × 10^−11^	OV	Caucasian	female	369	263
1.4445 (1.0980–1.8303)	8.83 × 10^−11^	STAD	Caucasian	male	153	240
1.1018 (0.8318–1.3940)	1.17 × 10^−10^	BRCA	Caucasian	female	653	263
1.1224 (0.8393–1.4289)	3.80 × 10^−10^	HNSC	Caucasian	male	300	240
1.2666 (0.9249–1.6396)	5.68 × 10^−9^	LUAD	Caucasian	female	165	263
0.9607 (0.6844–1.2734)	7.93 × 10^−8^	HNSC	Caucasian	female	120	263
0.9216 (0.6325–1.2450)	7.44 × 10^−7^	PRAD	Caucasian	male	127	240
0.8458 (0.5814–1.1452)	7.70 × 10^−7^	COAD	Caucasian	male	101	240
0.6438 (0.4208–0.8882)	6.00 × 10^−6^	SKCM	Caucasian	female	168	263
0.4797 (0.2712–0.7050)	2.71 × 10^−4^	LUAD	Caucasian	male	134	240
0.4689 (0.2634–0.6977)	3.82 × 10^−4^	LUSC	Caucasian	male	166	240
0.4903 (0.2585–0.7537)	1.12 × 10^−3^	LIHC	Asian	male	118	244

^1^ Log odds ratio, unit = per stand deviation. ^2^ Cancer abbreviations: BRCA—The Breast Cancer Gene; SKCM—Skin Cutaneous Melanoma; OV—Ovarian Serous Cystadenocarcinoma; STAD—Stomach Adenocarcinoma; HNSC—Head and Neck Squamous Cell Carcinoma; LUAD—Lung Adenocarcinoma; PRAD—Prostate Adenocarcinoma; COAD—Colon Adenocarcinoma; LUSC—Lung Squamous Cell Carcinoma; LIHC—Liver Hepatocellular Carcinoma. ^3^ The number of matched normal control were taken from International Genome Sample Resources (IGSR).

**Table 2:** Results from logistic regression assessing ROH’s cancer risk.

LogOR ^1^ (95% CI)	*p*	Cancer ^2^	Race	Gender	Cases	Controls ^3^
15.6383 (7.9048–23.5950)	0.0010	COAD	Caucasian	male	101	240
−0.2304 (−0.3658–−0.0969)	0.0048	OV	Caucasian	female	369	263
5.3577 (2.2553–8.5218)	0.0048	PRAD	Caucasian	male	127	240
−0.2839 (−0.4606–−0.1132)	0.0071	LUAD	Caucasian	female	165	263
−0.1797 (−0.2989–−0.0623)	0.0122	BRCA	Caucasian	female	653	263
−0.2557 (−0.4285–−0.0882)	0.0133	SKCM	Caucasian	female	168	263
8.1083 (2.2387–14.1885)	0.0253	HNSC	Caucasian	male	300	240
0.2548 (0.0708–0.4519)	0.0269	LIHC	Asian	male	118	244
7.1487 (1.0826–13.0103)	0.0422	LUSC	Caucasian	male	166	240
5.0918 (0.6647–9.4886)	0.0483	STAD	Caucasian	male	153	240
−0.2022 (−0.3918–−0.0282)	0.0670	BRCA	Black	female	163	342
−0.0873 (−0.2877–0.0971)	0.4534	HNSC	Caucasian	female	120	263
0.7471 (−0.0236–2.9058)	0.5483	SKCM	Caucasian	male	274	240
0.0625 (−0.1152–0.2397)	0.5614	LUAD	Caucasian	male	134	240

^1^ Log odds ratio, unit = per stand deviation. ^2^ Cancer abbreviations: COAD—Colon Adenocarcinoma; OV—Ovarian Serous Cystadenocarcinoma; PRAD—Prostate Adenocarcinoma; LUAD—Lung Adenocarcinoma; BRCA—The Breast Cancer Gene; SKCM—Skin Cutaneous Melanoma; HNSC—Head and Neck Squamous Cell Carcinoma; LIHC—Liver Hepatocellular Carcinoma; LUSC—Lung Squamous Cell Carcinoma; STAD—Stomach Adenocarcinoma. ^3^ The number of matched normal control were taken from IGSR.

**Table 3:** Association between global autozygosity and mutational signatures.

Effect	Stderr ^1^	Adusted p	Signature	Case	Predictor	Gender	Race	Cancer ^2^
0.2638	0.0387	4.62 × 10^−8^	SBS44	116	ROH	female	Caucasian	HNSC
0.3571	0.0679	3.02 × 10^−5^	SBS36	125	ROH	male	Caucasian	PRAD
0.8407	0.1970	0.0013	SBS9	277	HR_NonRef_	female	Caucasian	OV
1.3245	0.3223	0.0013	SBS18	277	HR_NonRef_	female	Caucasian	OV
1.0001	0.2359	0.0018	SBS42	164	ROH	male	Caucasian	LUSC
0.6597	0.1891	0.0070	SBS5	277	HR_NonRef_	female	Caucasian	OV
0.8939	0.2564	0.0070	SBS7c	277	HR_NonRef_	female	Caucasian	OV
2.9086	0.7533	0.0172	SBS7b	132	ROH	male	Caucasian	LUAD
−0.0885	0.0234	0.0206	SBS44	166	HR_NonRef_	female	Caucasian	SKCM
0.9714	0.3227	0.0280	SBS22	277	HR_NonRef_	female	Caucasian	OV
0.2439	0.0718	0.0453	SBS36	125	HR_NonRef_	male	Caucasian	PRAD

^1^ Standard error. ^2^ Cancer abbreviations: HNSC—Head and Neck Squamous Cell Carcinoma; PRAD—Prostate Adenocarcinoma; OV—Ovarian Serous Cystadenocarcinoma; LUSC—Lung Squamous Cell Carcinoma; LUAD—Lung Adenocarcinoma; SKCM—Skin Cutaneous Melanoma.

## Supplementary Materials

The following are available online at https://www.mdpi.com/2072-6694/12/12/3646/s1, Figure S1: Scatter plot between HR_NonRef_ and HR_Minor_, Figure S2: Comparison of HRNonRef and ROH between sex across all three races tested, Figure S3: ROC curves based on the cancer risk analysis in Table 1, Table S1: Cancer names and sample size, Table S2: List of mutational signatures.
