# Supplementary material for: Global Autozygosity Is Associated with Cancer Risk, Mutational Signature and Prognosis
Source: Cancers (Basel). 2020 Dec 4;12(12):3646. doi: 10.3390/cancers12123646 (PMC7761949; doi:10.3390/cancers12123646)
Supplement: Supplementary file 1 [file cancers-12-03646-s001.pdf]

Supplementary Materials: Global Autozygosity is Associated with Cancer Risk, Mutational Signature and Prognosis

Limin Jiang, Fei Guo, Jijun Tang, Shuguan Leng, Scott Ness, Fei Ye, Huining Kang, David C. Samuels and Yan Guo

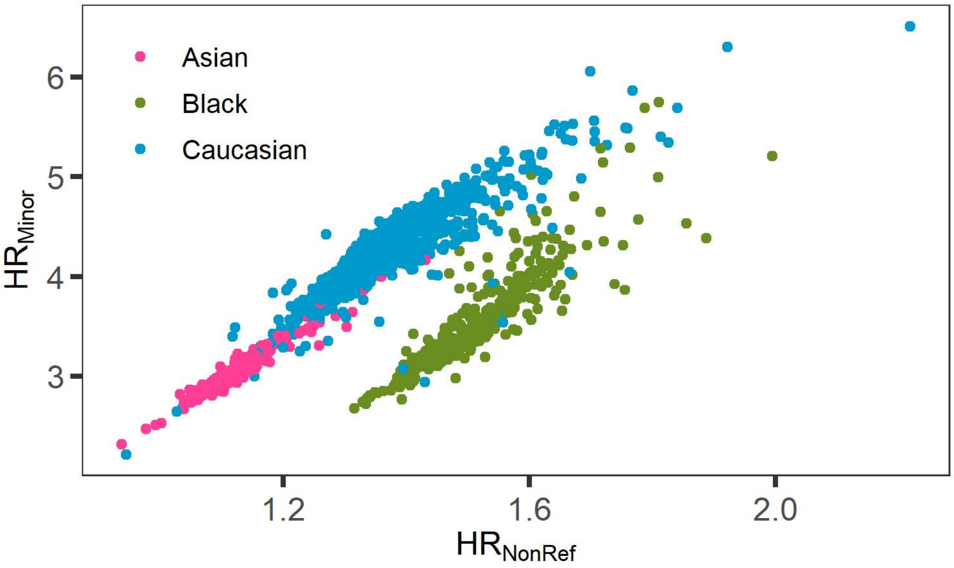

Figure S1. Scatter plot for HR_{NonRef} vs HR_{Minor}. Three clear clusters based on race can be observed. This suggests that HR is strongly associated with race.

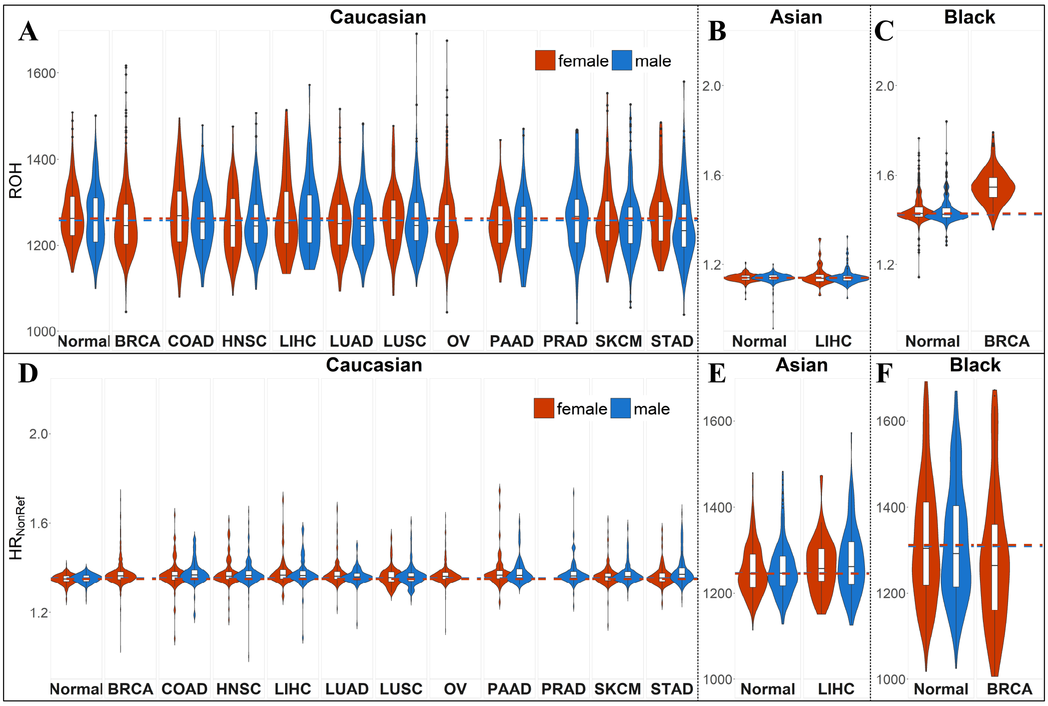

Figure S2. Comparison of HR_{NonRef} and ROH between sex across all three races tested. This is equivalent to Figure 3 from the main text without but removed data from sex chromosomes X and Y. The sex difference observed in Figure 3 were mostly gone.

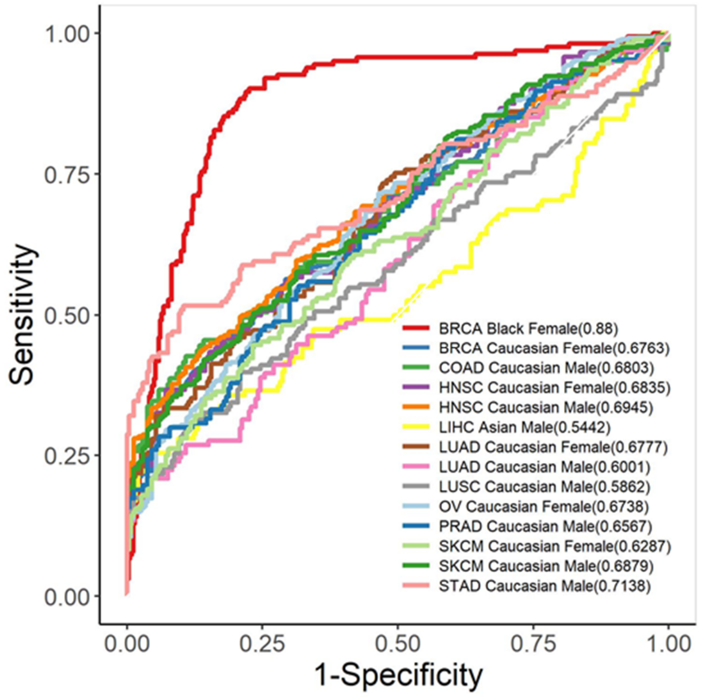

Figure S3. ROC curves based on the cancer risk analysis in Table 1.

Table S1. Cancer types encompassed in The Cancer Genome Atlas.

Cancer Abbreviation	Cancer Full Name	Sample Size
BRCA	Breast Invasive Carcinoma	883
COAD	Colon Adenocarcinoma	264
HNSC	Head and Neck Squamous Cell Carcinoma	476
LIHC	Liver Hepatocellular Carcinoma	299
LUAD	Lung Adenocarcinoma	340
LUSC	Lung Squamous Cell Carcinoma	250
OV	Ovarian Serous Cystadenocarcinoma	410
PAAD	Pancreatic Adenocarcinoma	143
PRAD	Prostate Adenocarcinoma	134
READ	Rectum Adenocarcinoma	84
SKCM	Skin Cutaneous Melanoma	455
STAD	Stomach Adenocarcinoma	319

Table S2. COSMIC mutational signatures (v3).

Signature	Etiology
**SBS1**	An endogenous mutational process initiated by spontaneous or enzymatic deamination of 5-methylcytosine to thymine which generates G:T mismatches in double stranded DNA. Failure to detect and remove these mismatches prior to DNA replication results in fixation of the T substitution for C.
**SBS2**	Attributed to activity of the AID/APOBEC family of cytidine deaminases on the basis of similarities in the sequence context of cytosine mutations caused by APOBEC enzymes in experimental systems. APOBEC3A is probably responsible for most mutations in human cancer, although APOBEC3B may also contribute (these differ in the sequence context two bases 5′ to the mutated cytosine, see 1,536 mutation classification signature extraction). SBS2 mutations may be generated directly by DNA replication across uracil or by error prone polymerases replicating across abasic sites generated by base excision repair removal of uracil.
**SBS3**	Defective homologous recombination-based DNA damage repair which manifests predominantly as small indels and genome rearrangements due to abnormal double strand break repair but also in the form of this base substitution signature.
**SBS4**	Associated with tobacco smoking. Its profile is similar to the mutational spectrum observed in experimental systems exposed to tobacco carcinogens such as benzo[a]pyrene. SBS4 is, therefore, likely due to direct DNA damage by tobacco smoke mutagens.
**SBS5**	**Unknown** SBS5 mutational burden is increased in bladder cancer samples with ERCC2 mutations and in many cancer types due to tobacco smoking.

**SBS6**	SBS6 is associated with defective DNA mismatch repair and is found in microsatellite unstable tumours.
**SBS7a**	SBS7a/SBS7b/SBS7c/SBS7d are found in cancers of the skin from sun exposed areas and are thus likely to be due to exposure to ultraviolet light. SBS7a may possibly be the consequence of just one of the two major known UV photoproducts, cyclobutane pyrimidine dimers or 6-4 photoproducts. However, there is currently no evidence for this hypothesis and it is unclear which of these photoproducts may be responsible for SBS7a.
**SBS7b**	SBS7a/SBS7b/SBS7c/SBS7d are found in cancers of the skin from sun exposed areas and are likely to be due to exposure to ultraviolet light. SBS7b may possibly be the consequence of just one of the two major known UV photoproducts, cyclobutane pyrimidine dimers or 6-4 photoproducts. However, there is no evidence for this hypothesis and it is unclear which of these photoproducts may be responsible for SBS7b.
**SBS7c**	SBS7a/SBS7b/SBS7c/SBS7d are found in cancers of the skin from sun exposed areas and are likely to be due to exposure to ultraviolet light. SBS7c is possibly the consequence of translesion DNA synthesis by enzymes with propensity to insert T, rather than A, opposite ultraviolet induced thymidine and cytidine photodimers. The preponderance of T>A rather than T>C mutations may reflect the heavier burden of thymidine compared to cytidine dimers induced by UV light.
**SBS7d**	SBS7a/SBS7b/SBS7c/SBS7d are found in cancers of the skin from sun exposed areas and are likely to be due to exposure to ultraviolet light. SBS7d is possibly the consequence of translesion DNA synthesis by error-prone polymerases with greater propensity to insert G, rather than A, opposite UV light induced thymidine and cytidine photodimers.
**SBS8**	**Unknown**
**SBS9**	May be due in part to mutations induced during replication by polymerase eta as part of somatic hypermutation in lymphoid cells.
**SBS10a**	Polymerase epsilon exonuclease domain mutations.
**SBS10b**	Polymerase epsilon exonuclease domain mutations.
**SBS11**	SBS11 exhibits a mutational pattern resembling that of alkylating agents. Patient histories indicate an association between previous treatment with the alkylating agent temozolomide and SBS11 mutations.
**SBS12**	**Unknown**
**SBS13**	Attributed to activity of the AID/APOBEC family of cytidine deaminases on the basis of similarities in the sequence context of cytosine mutations caused by APOBEC enzymes in experimental systems. APOBEC3A is probably responsible for most mutations in human cancer, although APOBEC3B may also contribute (these differ in the sequence context two bases 5′ to the mutated cytosine, see 1536 mutation classification signature extraction). SBS13 mutations are likely generated by error prone polymerases (such as REV1) replicating across abasic sites generated by base excision repair removal of uracil.
**SBS14**	Concurrent polymerase epsilon mutation and defective DNA mismatch repair.
**SBS15**	Defective DNA mismatch repair.
**SBS16**	**Unknown**
**SBS17a**	**Unknown**
**SBS17b**	**Unknown**
**SBS18**	Possibly damage by reactive oxygen species.
**SBS19**	**Unknown**
**SBS20**	Concurrent POLD1 mutations and defective DNA mismatch repair.
**SBS21**	DNA mismatch repair deficiency.
**SBS22**	Aristolochic acid exposure. Found in cancer samples with known exposures to aristolochic acid and the pattern of mutations exhibited by the signature is consistent with that observed in experimental systems of aristolochic acid exposure.
**SBS23**	**Unknown**
**SBS24**	Aflatoxin exposure. SBS24 has been found in cancer samples with known exposures to aflatoxin and the pattern of mutations exhibited by the signature is consistent with that observed in experimental systems exposed to aflatoxin.
**SBS25**	**Unknown** However, some Hodgkin's cell line samples in which the signature has been found were from patients exposed to chemotherapy and it is possible that SBS25 is due to chemotherapy treatment.
**SBS26**	Defective DNA mismatch repair.
**SBS28**	**Unknown**
**SBS29**	SBS29 has been found in cancer samples from individuals with a tobacco chewing habit.
**SBS30**	SBS30 is due to deficiency in base excision repair due to inactivating mutations in NTHL1.
**SBS31**	Prior chemotherapy treatment with platinum drugs.
**SBS32**	Prior treatment with azathioprine to induce immunosuppression. Associated mutation classes and signatures
**SBS33**	**N/A**
**SBS34**	**Unknown**
**SBS35**	Prior chemotherapy treatment with platinum drugs.
**SBS36**	Defective base excision repair, including DNA damage due to reactive oxygen species, due to biallelic germline or somatic MUTYH mutations.
**SBS37**	**Unknown**

**SBS38**	**Unknown** Found only in ultraviolet light associated melanomas suggesting potential indirect damage from UV-light.
**SBS39**	**Unknown**
**SBS40**	**Unknown**
**SBS41**	**Unknown**
**SBS42**	Occupational exposure to haloalkanes.
**SBS44**	Defective DNA mismatch repair.
**SBS84**	Activity of activation-induced cytidine deaminase (AID).
**SBS85**	Indirect effects of activation-induced cytidine deaminase (AID) induced somatic mutagenesis in lymphoid cells.

by the authors. Licensee MDPI, Basel, Switzerland. This article is an open access article distributed under the terms and conditions of the Creative Commons Attribution (CC BY) license (http://creativecommons.org/licenses/by/4.0/).
